# I feel blue– teacher, can you help me? A study on the effect of digital literacies on language learners’ technostress, on-line engagement, autonomy, and academic success

**DOI:** 10.1186/s40359-024-01637-5

**Published:** 2024-03-13

**Authors:** Xiuxia Feng, Huying Liu

**Affiliations:** 1 School of General Education and Foreign languages, Shanghai Lida University, 201608 Shanghai, China; 2 SWAN College, Central South University of Forestry & Technology, 410211 Changsha, China; 3 Graduate School, Stamford International University, 10250 Bangkok, Thailand

**Keywords:** Academic success, Autonomy, Digital Literacy, Online Engagement, Technostress

## Abstract

This phenomenological study explored the experiences of language learners in the digital age, specifically investigating the intersection of digital literacy, technostress, online engagement, autonomy, and academic success. Twenty participants, selected through purposive sampling, shared Chinese as their native language and were between 18 and 20 years old, with five participants being female. Employing interviews and document analysis, the study aimed to understand the subjective meanings, emotions, and perceptions associated with these phenomena. The findings revealed the multifaceted nature of technostress, the crucial role of digital literacy in shaping online engagement and autonomy, and the nuanced impact on academic success. These qualitative insights contribute to a deeper understanding of the complex relationships in the digital language learning landscape. The study has implications for educators, materials developers, syllabus designers, and policy-makers, providing practical insights to enhance language learning experiences in the digital era. Future research may further explore specific dimensions uncovered in this study to adapt educational practices to the evolving digital terrain.

## Introduction

Online education is no longer a recent development, as it has progressively gained traction in universities worldwide since the final decade of the twentieth century. It emerged as an appealing choice for universities aiming to broaden their reach by providing flexible alternatives for students seeking courses without the need for relocation [1–2]. Simultaneously, online learning and teaching allowed universities to provide enhanced flexibility to staff and engage those with valuable expertise who were physically distant but accessible through remote collaboration. The study aims to investigate how digital literacy influences language learners’ experiences in online education, particularly regarding technostress, online engagement, autonomy, and academic success.

In recent years, the prevalence of online education has surged, driven by advancements in technology and an increasing demand for flexible learning options. This shift towards online learning has been further accelerated by global events such as the COVID-19 pandemic, which necessitated the rapid adoption of digital platforms for education. While online education offers numerous benefits, including accessibility and flexibility, it also presents unique challenges for learners, educators, and institutions alike. Challenges such as technostress, digital divide, and maintaining student engagement in virtual settings have become increasingly prevalent. Moreover, the transition to online learning has highlighted disparities in digital literacy skills among learners, further exacerbating these challenges. Therefore, understanding the complex interplay between digital literacy and the experiences of language learners in the online education domain is paramount in addressing these challenges and ensuring the effectiveness and inclusivity of digital learning environments.

As the educational landscape evolved, the role of digital literacy skills became increasingly pivotal. These skills encompass the competence to access information, operate, and communicate safely and efficiently through digital technologies and online resources [3–5]. Digital literacy not only guides us in understanding the purpose and usage of technology but also highlights the potential benefits we can derive from it in our daily lives [5–6].

In 1984, the term technostress emerged, referring to a condition attributed to an individual’s challenges in coping with technology [7]. Will and Rosen characterized technostress as encompassing any adverse impact on attitudes, thoughts, behaviors, or physiological well-being directly or indirectly induced by technology [8]. Recently, a prominent definition of technostress has emerged, characterizing it as user stress arising from multitasking in the application and continuous communication, repeated information, frequent system improvements, resulting uncertainty, ongoing re-learning, functional insecurity, and technical problems associated with the use of information and communication technologies within organizations [9].

This growing awareness of technostress, coupled with the increasing reliance on online education [1–2], sets the stage for understanding the challenges that language learners may face in the digital realm. However, one of the widely accepted definitions in the literature describes technostress as the phenomenon experienced by end-users in organizations due to their use of information and communication technologies [10]. Recognizing the profound impact of technostress on individuals navigating online education, our study delves into its effects on language learners, exploring dimensions such as technostress’s influence on online engagement, autonomy, and ultimately, academic success.

Another construct worthy of attention is online engagement which is a complex concept that involves diverse activities such as commenting, sharing, liking, and contributing user-generated content. These activities aim to facilitate meaningful interactions and information exchanges in the digital domain [11–12]. Elements of online engagement have been introduced, encompassing indicators related to student beliefs, attitudes, and behaviors. This aids researchers and educators in evaluating the effectiveness of online courses in engaging students. The significance of successful online engagement is underscored by psychosocial and structural factors, emphasizing the roles of peer community, teacher engagement, workload, and course design [11]. While the term ‘engagement’ is challenging to define in educational technology and online learning, it is recognized as involving effort, commitment, active learning, and supportive environments [13–14].

Learner autonomy refers to the capability to take charge of one’s own affairs and to operate effectively in situations where the learner assumes complete responsibility for all decisions related to their learning and the subsequent execution of those decisions [15–16]. An autonomous individual possesses the ability to independently make and execute choices governing their actions [16]. Individuals with autonomy in learning view themselves as having authority, are driven by intrinsic motivation, exhibit self-confidence, and demonstrate a proficiency for engaging in active and self-directed learning [17].

Achieving academic success is a multifaceted endeavor encompassing a learner’s ability to meet educational goals, acquire knowledge, and demonstrate proficiency in various subjects. Measurement of academic success traditionally involves performance metrics such as grades, exam scores, and overall grade point average [18]. However, in the context of online education and the digital era, the assessment landscape has expanded to include factors like online participation, digital engagement, and proficiency in utilizing digital tools [19]. Several studies have shed light on the evolving nature of academic success in the digital age. For instance, [20] found a positive correlation between students’ digital literacy skills and their academic performance, emphasizing the role of digital literacy in navigating online learning environments effectively. Moreover, recent research [21] indicated that learners with higher digital literacy levels tend to demonstrate greater adaptability to digital assessment methods, positively impacting their overall academic success.

The proliferation of online education, driven by its accessibility and flexibility, has reshaped the educational landscape globally. While the advantages of online learning are evident, the confluence of digital literacy skills and the emerging phenomenon of technostress poses challenges for language learners engaged in this digital realm. As educational institutions increasingly rely on online platforms [1–2], the escalating awareness of technostress raises concerns about its implications for language learners navigating digital environments. Technostress, characterized by adverse impacts on attitudes, thoughts, behaviors, and physiological well-being induced by technology [8–9], presents a nuanced challenge for language learners in online education [10]. This study seeks to address the gap in understanding the intricate relationship between technostress, online engagement, learner autonomy, and their collective impact on the academic success of language learners. Specifically, we aim to explore the dimensions of technostress and its influence on online engagement, autonomy, and academic success in the digital learning landscape. Furthermore, we seek to examine how digital literacy skills, recognized for their role in effective online navigation [20–21], may mediate or exacerbate the effects of technostress on language learners’ educational outcomes. In doing so, this research contributes to the ongoing discourse on the challenges posed by the intersection of technology, language learning, and academic success in the dynamic context of online education.

The objectives of our research are manifold, aiming to comprehensively understand the intricate dynamics between digital literacy, technostress, online engagement, learner autonomy, and academic success among language learners in online education.

Firstly, we seek to assess the impact of digital literacy on learners’ technostress experiences. This involves investigating whether varying levels of digital literacy skills among language learners are correlated with differences in their encounters with technostress. Additionally, we aim to examine how specific components of digital literacy, such as information access, communication efficiency, and technology operation, contribute to or alleviate the prevalence of technostress.

Secondly, our study aims to examine the relationship between digital literacy and learners’ online engagement activities. This includes exploring the extent to which language learners’ digital literacy skills influence their online interactions, encompassing activities such as commenting, sharing, liking, and contributing user-generated content. We also aim to analyze how digital literacy shapes the quality and depth of these online engagements, evaluating the role of learners’ digital proficiency in fostering meaningful interactions in the digital learning environment.

Furthermore, we aim to evaluate the impact of digital literacy on learners’ autonomy in the online learning environment. This objective involves investigating the correlation between language learners’ digital literacy levels and their capacity for autonomous decision-making. We will explore how digital literacy skills contribute to learners’ sense of authority, intrinsic motivation, self-confidence, and proficiency in engaging in active and self-directed learning.

Lastly, our research seeks to assess the influence of digital literacy on learners’ academic success. This involves exploring the relationship between language learners’ digital literacy proficiency and their academic achievements, considering traditional metrics such as grades, exam scores, and overall grade point average. Additionally, we will investigate whether learners with higher digital literacy levels demonstrate greater adaptability to digital assessment methods, ultimately impacting their overall academic success. Through these objectives, our research endeavors to provide comprehensive insights into the multifaceted dynamics of digital literacy in the online learning landscape. Thus, we address the following research questions:

1. How does digital literacy affect learners’ technostress?
2. How does digital literacy affect learners’ online engagement?
3. How does digital literacy affect learners’ autonomy?
4. How does digital literacy affect learners’ academic success?

The significance of this study lies in its comprehensive exploration of the intricate interplay between digital literacy, technostress, online engagement, learner autonomy, and academic success among language learners in the evolving landscape of online education. This research holds several key implications and contributions.

Understanding the impact of digital literacy on language learners can inform the design and implementation of educational practices. Insights gained from this study can guide educators in tailoring digital literacy interventions to enhance students’ experiences and outcomes in online learning environments.

As the awareness of technostress grows, this study contributes to a nuanced understanding of its implications for language learners. By identifying specific areas of technostress related to digital literacy, educators and institutions can develop targeted interventions to alleviate stressors and enhance the overall well-being of learners.

Examining the relationship between digital literacy and online engagement provides valuable insights into effective strategies for fostering meaningful interactions in the digital domain. Educators can use these findings to optimize online engagement activities, ultimately enhancing the overall quality of the learning experience.

The investigation into the role of digital literacy in learner autonomy contributes to the empowerment of language learners. Understanding how digital literacy skills foster autonomy can guide the development of educational practices that nurture self-directed learning and decision-making among students.

The study’s exploration of the relationship between digital literacy and academic success adds depth to the understanding of learners’ performance in the digital age. Institutions can use these insights to develop targeted interventions that leverage digital literacy skills to enhance academic achievements in online learning environments.

This research contributes to the scholarly discourse by addressing a gap in the literature concerning the multifaceted impact of digital literacy on language learners in online education. The findings will provide a foundation for future research endeavors, facilitating a deeper understanding of the complex dynamics within the digital learning environment. In essence, the significance of this study extends beyond the immediate context of language learners in online education, offering valuable insights and practical implications for educators, institutions, and researchers navigating the dynamic intersection of digital literacy and language education in the digital era.

This study offers a novel contribution to the existing literature by adopting a phenomenological approach to explore the experiences of language learners in the digital age. Unlike many quantitative studies focusing solely on digital literacy or technostress, this qualitative investigation delves deeply into the subjective meanings, emotions, and perceptions associated with these phenomena. By immersing itself in the lived experiences of language learners, the study tries to uncover nuanced insights that complement and expand upon existing quantitative research, providing a holistic understanding of the challenges and opportunities presented by the digital landscape in language education.

## Literature Review

### Theoretical background

#### Digital Literacy

Digital literacy, commonly known as literacy in the digital age, has entrenched itself in educational discourse [22]. Despite its early acknowledgment, defining digital literacy proves challenging due to its expansive and continually evolving nature [22–23]. Digital literacy is a complex concept that necessitates an understanding of practices, attitudes, and behaviors specific to particular digital environments [22]. In essence, it is context-dependent and varies based on the technological landscape and digital tools in use [24]. Within Australian education, digital literacy encompasses knowledge and skills enabling students to proficiently create, manage, communicate, investigate data, and collaborate [25]. This definition reflects the diverse abilities required to navigate and utilize digital technologies across various educational facets and beyond. The multifaceted nature of digital literacy underscores the need for Pre-Service Teachers (PSTs) to continually adapt to evolving technologies [26]. In the swiftly changing digital realm, PSTs must stay abreast of the latest tools and systems to effectively engage with information and communication technologies. Digital literacy also entails critically identifying and utilizing digital devices and systems while ensuring one’s safety in digital environments [25]. This aspect underscores the significance of responsible and ethical digital practices to safeguard oneself and others when using technology in teaching and learning. Recent research posits that digital literacy is vital not only for utilizing technology in teaching but also for acquiring other essential competencies crucial for active participation in modern society and the economy [27]. As technology permeates various domains like communication, employment, research, and information access, digital literacy has become indispensable in everyday life. Developing digital literacy skills empowers individuals to navigate the digital landscape effectively and seize the opportunities it presents.

### Technostress

The initial effort to define technostress was initiated by Brod [28], who characterized it as a contemporary ailment arising from the inability to adapt healthily to emerging computer technologies. Subsequently, [29] described technostress as an elevated state experienced by individuals extensively using computers for work-related tasks. Recent investigations [30–32] have expanded this definition to include any stress encountered by users due to the utilization of information and communications technologies.

The exploration of the technostress concept has been limited in the literature, with few studies addressing it [31, 33]. Although the government and industrial sectors have engaged with this idea [34], its examination in the educational sector is comparatively scarce [31]. Some investigations have specifically concentrated on university professors, revealing that they undergo increased stress while adapting to new and more demanding online assignments [35]. The ramifications of technostress reach into individuals’ social, physiological, and psychological well-being, potentially affecting sleep patterns and social interactions [36].

### Online Engagement

Online engagement is a multifaceted concept, involving a variety of activities such as commenting, sharing, liking, and contributing user-generated content. These endeavors are geared towards fostering meaningful interactions and facilitating the exchange of information in the digital domain [11–12]. The components of online engagement have been delineated, covering indicators linked to student beliefs, attitudes, and behaviors. This framework assists researchers and educators in gauging the efficacy of online courses in engaging students. The importance of successful online engagement is accentuated by a combination of psychosocial and structural factors, highlighting the pivotal roles played by peer community, teacher engagement, workload, and course design [11]. Despite the inherent challenge in precisely defining the term ‘engagement’ within the realms of educational technology and online learning, it is acknowledged to encompass attributes such as effort, commitment, active learning, and supportive environments [13–14].

Examining online engagement is a relatively recent focus within the research domain, and there are limited frameworks available for assessing the engagement of online students. A noteworthy study [37] revealed that students’ engagement in an online learning environment, encompassing behavioral, cognitive, and emotional aspects, led to a more profound comprehension of subject content. Subsequently, [38] introduced a model linking digital literacy, attitude, and efficacy, later expanding it to incorporate online engagement. However, their definition of online engagement was constrained, covering only social and academic (cognitive) interactions [12]. contributed to this area by expanding the definition of online engagement within higher education, introducing three additional constructs: behavioral engagement, collaborative engagement, and emotional engagement.

### Autonomy

Autonomy represents one of the foundational psychological needs outlined in the Self-Determination Theory (SDT) [39–41]. The satisfaction of these needs serves as an inherently motivating factor with significant implications for personal development and well-being (ibid.). In this context, autonomy pertains to the engagement in actions aligned with one’s personal beliefs, genuine interests, and values. The degree of autonomy associated with regulating behavior plays a critical role in influencing performance, persistence, and overall well-being. As a result, autonomy emerges as a pivotal factor in the regulation of motivation [39–41].

Researchers have underscored the connection between autonomy and motivation in the journey of second language (L2) acquisition (e.g., [42–45]), particularly within online learning environments [46]. Autonomy stands out as a more influential predictor of proficiency compared to language anxiety and motivation in this context [47]. The digital learning landscape is acknowledged as both demanding [48] and holding the potential to enhance learners’ autonomy. The affordances span from providing access to resources anytime, anywhere to heightening students’ awareness of the learning process [49] and cultivating positive attitudes toward autonomous learning [50]. However, a cautious note is sounded regarding the potential risk of technology fostering a misguided sense of progress in students [51].

### Academic success

Attaining academic success is a multifaceted pursuit involving a learner’s capacity to achieve educational objectives, acquire knowledge, and exhibit proficiency across various subjects. Traditionally, the evaluation of academic success has relied on performance metrics like grades, exam scores, and overall grade point average [18]. However, with the advent of online education and the digital era, the assessment paradigm has expanded to encompass factors such as online participation, digital engagement, and proficiency in utilizing digital tools [19]. Numerous studies have illuminated the evolving nature of academic success in the digital age. For instance, [20] identified a positive correlation between students’ digital literacy skills and their academic performance, underscoring the pivotal role of digital literacy in effectively navigating online learning environments. Furthermore, recent research [21] suggested that learners with heightened digital literacy levels exhibit greater adaptability to digital assessment methods, thereby positively influencing their overall academic success.

### Empirical background

Over the past decade, scholars have increasingly emphasized the importance of integrating technologies into L2 education, highlighting the need for L2 educators to attain digital literacy. Despite this, there has been a lack of research exploring the connection between English as a foreign language (EFL) students’ digital literacy, technostress, and academic productivity [52]. addressed this gap, utilizing three validated questionnaires to investigate the impact of digital literacy on Chinese EFL students. The results, obtained through structural equation modeling (SEM) and multiple regression, indicated that digital literacy significantly influenced both technostress and academic productivity, predicting 77% and 61% of changes, respectively.

Hussain et al. [53] investigated the relationship between digital literacy, performance expectancy, and the intention to use digital technologies in technology educational institutes. Employing Technostress as a moderating variable, a questionnaire survey was conducted with 283 students in Lahore, Punjab, Pakistan, who were enrolled in various 3-year Diploma programs. Out of these, 260 valid responses were analyzed using Partial Least Squares SEM. The findings indicated a significant influence of performance expectancy on the intention to use Digital Literacy and Digital Technologies, with Technostress acting as a moderator. Additionally, Performance expectancy served as a mediator, showing a significant impact on the intention to use digital technologies.

Martínez-Navalón et al. [54] examined the relationship between digital knowledge management, technostress, and organizational sustainability through a literature review covering key dimensions. A questionnaire, comprising respondent classification, knowledge management, technostress, and sustainability sections, garnered 454 valid responses. Findings revealed a direct, positive link between knowledge management and technostress, indicating that higher knowledge management levels in firms led to increased technostress among employees. The study also confirmed a significant association between knowledge management and organizational sustainability. Gender was found to be a non-significant moderator, showing no discernible differences in the reported relationships. Descriptive analysis and structural equation modeling with the partial least squares method were employed for the research.

Getenet et al. [55] investigated the relationships among students’ online engagement, digital technology attitude, digital literacy, and self-efficacy within an Australian regional university. Data from 110 first-year students were collected through a field survey, and AMOS 28 was used for measurement and structural model path analysis. The study examined the impact of student attitudes and digital literacy on self-efficacy, followed by assessing the effects of self-efficacy on five dimensions of online engagement: social, collaborative, cognitive, behavioral, and emotional. The results indicated that positive attitudes and digital literacy significantly contributed to self-efficacy, positively influencing the dimensions of online engagement. The findings emphasize the importance of incorporating various engagement elements when designing and facilitating online, blended, or technology-enhanced courses in higher education. The study underscores the role of student attitudes and digital literacy in fostering self-efficacy and enhancing online learning engagements.

To provide a high standard of education for every student, educators need to possess proficient knowledge and abilities in utilizing digital technologies for instructional purposes [56]. assessed the outlook of PSTs in an online context regarding their digital attitude, efficacy, literacy, engagement, and understanding of digital technologies. The research established notable links between PSTs’ self-efficacy and their outlooks toward digital technologies, digital literacies, and learner engagement.

The rapid evolution of digital technologies and the internet has transformed access to language resources and language learning methodologies. High school students are increasingly exposed to digitalized learning activities, shaping their independent learning approaches. Despite the prevalence of Autonomous Learning and Digital Literacy, limited research has explored the relationship between the two. Drawing on the responses of 224 high school students to questionnaires, [57] delved into their digital literacy across five dimensions, including knowledge acquisition, and their autonomous learning situations from two perspectives. The findings revealed that participants possess a certain level of digital literacy, express a high desire to learn, and exhibit a medium level of self-management capacity. However, no correlation was identified between students’ digital literacy levels and their levels of autonomy as learners.

Kara and Mede [58] investigated the impact of online education on digital literacy and autonomy readiness in a Turkish PST education program. The study, involving 49 participants from the Department of English Language Teaching, utilized pre- and post-test results from the Digital Literacy Scale and Learner Autonomy Readiness Questionnaire, along with semi-structured interviews. The findings indicated an improvement in both digital literacy and learner autonomy readiness post-online education, with positive attitudes towards technology and autonomous learning revealed through interviews. Despite this, a non-significant correlation was observed between digital literacy and learner autonomy readiness.

Naz et al. [59] investigated the impact of digital literacy on the academic performance of university-level students in Pakistan. Conducted as a descriptive study, a survey collected responses from 120 students in public sector universities, which were analyzed using Mean-difference statistics. The findings indicated that students possessing digital knowledge or technological skills performed better than those without such skills. Additionally, female students with digital skills outperformed their male counterparts at a similar digital literacy level. The study highlighted the importance of teaching all university-level students how to effectively use library computers for research purposes.

Lopez [60] explored the connection between digital literacy and academic performance in an online learning high school program catering to underprivileged students. The research proposed viewing digital literacy as a construct with various components, ranging from basic access to technology to advanced skills and attitudes crucial for success in online learning. Utilizing path analysis, the study tested a three-stage model, assessing the effects of variables within the digital literacy construct and their impact on academic performance. The model included stages related to access to technology, general digital skills (covering motivation, knowledge, technology usage frequency, and diversity), and context-specific skills for online distance learning. The findings revealed that improved access to technology positively influenced academic performance through increased Internet use for learning purposes, mediated by enhanced digital and academic skills. Another noteworthy finding indicated that better access conditions led to increased use of social networks, which had both positive effects (familiarity with the Internet) and negative effects (potential time diversion from learning).

These studies collectively highlight the intricate relationship between digital literacy, technostress, online engagement, learner autonomy, and academic success across various educational settings. They reveal the predictive power of digital literacy in shaping technostress and academic productivity, emphasizing its significance in educational practices and organizational sustainability. Additionally, they underscore the importance of fostering positive attitudes and digital competencies among students and educators to enhance self-efficacy, engagement, and academic performance in online learning environments. These findings contribute valuable insights into the evolving landscape of digital education, informing strategies for optimizing learning outcomes in the digital age.

Despite the increasing emphasis on integrating technologies into L2 education over the past decade and the recognition of the crucial role of digital literacy for language educators, a notable gap exists in research exploring the nexus between EFL students’ digital literacy, technostress, and academic productivity. Previous studies have laid the foundation, revealing significant correlations between digital literacy, technostress, and academic outcomes among diverse populations such as Chinese EFL students, technology education institute students in Pakistan, and university-level students in Pakistan. However, there is a distinct dearth of investigations focusing specifically on high school students and PSTs, with limited exploration of the relationship between autonomous learning and digital literacy. Moreover, while some studies have shown positive connections between digital literacy and academic performance, others have highlighted nuanced relationships that necessitate further examination. Given the evolving landscape of digital learning and the transformative effects of the internet on language resources and methodologies, this study seeks to address these gaps by delving into the intricate relationships between digital literacy, technostress, online engagement, learner autonomy, and academic success, contributing valuable insights to the intersection of technology, language learning, and educational outcomes.

## Method

### Design

This study adopted a phenomenological research design to delve into the lived experiences and perceptions of language learners in the realm of digital literacy, technostress, online engagement, learner autonomy, and academic success. Phenomenology was chosen as the guiding approach due to its suitability for exploring the subjective meanings and interpretations individuals attribute to their experiences, offering an in-depth understanding of the phenomena under investigation. The study involved a qualitative inquiry, relying on interviews and document analysis to gather rich and detailed data from language learners engaged in online education.

The phenomenological approach was chosen for its suitability in exploring the subjective meanings, emotions, and perceptions associated with the experiences of language learners in the digital realm. Unlike quantitative methods that focus on numerical data, phenomenology delves into the lived experiences of individuals, providing rich and detailed insights into their perspectives. This approach allowed for a deep and nuanced exploration of the complex interplay between digital literacy, technostress, online engagement, autonomy, and academic success, capturing the essence of participants’ experiences in their own words.

The rigor of the study was ensured through several key measures. Firstly, the use of a phenomenological approach allowed for in-depth exploration of participants’ lived experiences, providing rich qualitative data that captured the essence of their encounters with digital literacy, technostress, online engagement, autonomy, and academic success. Secondly, member checking and peer debriefing were employed to enhance the credibility and trustworthiness of the findings. Participant feedback during member checking sessions helped validate the interpretations of their experiences, ensuring that the analysis accurately reflected their perspectives. Similarly, peer debriefing involved discussions with colleagues to critically examine the research process and findings, offering diverse viewpoints and insights that strengthened the overall rigor of the study. Additionally, the triangulation of data sources, including semi-structured interviews and document analysis, further bolstered the study’s credibility by providing multiple perspectives on the phenomena under investigation. Finally, transparent and systematic data analysis procedures were followed, including thorough coding and thematic analysis, to ensure consistency and rigor in the interpretation of the findings. Overall, these methodological strategies contributed to the robustness of the study and enhanced its credibility and trustworthiness.

### Participants

In this phenomenological study conducted in the context of online language education, 20 participants were selected through purposive sampling. All participants shared Chinese as their native language and were between 18 and 20 years old. Among them, five participants were female, while the remaining were male. None of the participants had ever visited an English-speaking country. The research focused on understanding the unique perspectives and experiences of these participants in the online education setting, with an emphasis on exploring their digital literacy levels, technostress encounters, online engagement practices, learner autonomy, and academic success. Ethical considerations, including informed consent and confidentiality, were prioritized throughout the research process.

The inclusion criteria for participants were meticulously established to ensure the selection of individuals meeting specific characteristics relevant to the study’s objectives. Participants were required to be language learners enrolled in online education programs, specifically focusing on English as a foreign language (EFL) studies. Furthermore, they were restricted to individuals aged between 18 and 20, native speakers of Chinese, and exhibiting varying levels of digital literacy. These criteria were crucial for targeting a specific demographic—language learners navigating the digital landscape—and for capturing diverse experiences and viewpoints pertinent to the research inquiries. Purposive sampling was employed to select participants capable of providing comprehensive insights into the interplay of digital literacy, technostress, online engagement, autonomy, and academic success, thereby enriching the study’s depth and breadth of.

### Instruments

In this study, data collection employed interviews and document analysis as the primary instruments. Semi-structured interviews were conducted to explore participants’ experiences, perceptions, and insights regarding digital literacy (modified version of [61]), technostress (modified version of [62]), online engagement (modified version of [63]), learner autonomy (modified version of [64]), and academic success (modified version of [65]) in the context of online language education. Additionally, document analysis involved the examination of relevant documents such as academic records and online engagement metrics to triangulate and complement the interview data. To ensure trustworthiness, the credibility of the study was enhanced through prolonged engagement with participants, member checking, and peer debriefing. Member checking involved sharing summaries of participants’ experiences with them to confirm accuracy. Peer debriefing involved discussions with colleagues to gain alternative perspectives and insights. Transferability was addressed by providing a detailed description of the study context, participants, and methodology to facilitate the applicability of findings to similar settings. Confirmability was assured by maintaining an audit trail, documenting decision-making processes, and engaging in reflexive practices to minimize researcher bias throughout the study.

### Data Collection procedures

In this study, data collection involved a two-fold approach: semi-structured interviews and document analysis. Semi-structured interviews were conducted with the 20 participants selected through purposive sampling. Each participant was individually interviewed, and open-ended questions were posed to explore their experiences with digital literacy, technostress, online engagement, learner autonomy, and academic success in the online language education context. The interviews were audio-recorded to ensure accuracy during subsequent analysis.

Simultaneously, document analysis was performed by examining relevant documents, including academic records and online engagement metrics. Academic records provided insights into participants’ academic success, while online engagement metrics offered data on their interactions and participation in the online learning environment.

The process of document analysis involved a systematic review and interpretation of academic records and online engagement metrics to extract relevant information pertaining to the study’s objectives. Firstly, academic records, including transcripts, grades, and exam scores, were meticulously examined to identify patterns and trends related to participants’ academic success. This examination involved comparing the academic performance of participants with varying levels of digital literacy to discern any correlations between digital literacy and academic outcomes. Secondly, online engagement metrics, such as participation in discussion forums, contributions to collaborative tasks, and usage of digital resources, were scrutinized to assess participants’ levels of engagement in the online learning environment. This analysis included quantifying and categorizing participants’ online interactions to determine the extent to which digital literacy influenced their engagement behaviors. Overall, the document analysis process aimed to triangulate and complement the qualitative insights obtained from interviews, providing a comprehensive understanding of the relationship between digital literacy, online engagement, and academic success among language learners in the context of online education.

To conduct the semi-structured interviews, a predetermined set of open-ended questions was developed, addressing key aspects related to digital literacy, technostress, online engagement, learner autonomy, and academic success. The interviews were carried out in a conversational manner, allowing participants to express their thoughts and experiences freely. The document analysis process involved systematically reviewing and interpreting the content of academic records and online engagement metrics to extract relevant information pertaining to the study’s objectives.

To enhance the rigor of the study, efforts were made to establish trustworthiness through prolonged engagement with participants, member checking, and peer debriefing. Member checking involved sharing interview summaries with participants for their validation and input. Peer debriefing included discussions with colleagues to obtain diverse perspectives and ensure the credibility of the data. Throughout the data collection process, ethical considerations were prioritized, including obtaining informed consent, ensuring participant confidentiality, and maintaining the integrity of the research.

Member checking and peer debriefing were integral components of the research process, contributing to the rigor and credibility of the study. During member checking, participants were provided with summaries or excerpts of their interview transcripts to review for accuracy and completeness. Their feedback allowed us to ensure the trustworthiness of the data by confirming the alignment between participants’ perspectives and the interpretations derived from the analysis. Participant feedback also provided valuable insights into potential areas of clarification or additional exploration, guiding subsequent data collection and analysis efforts.

Peer debriefing involved discussions with colleagues or experts in the field who were not directly involved in the study. These discussions served multiple purposes, including validating the interpretations drawn from the data, challenging assumptions, and generating alternative perspectives. Colleagues offered critical feedback on the research process, methodology, and findings, prompting reflexivity and enhancing the overall quality of the study. Insights gained from peer debriefing discussions helped refine the analysis, deepen the interpretation of themes, and ensure the robustness of the study’s conclusions. Additionally, peer debriefing provided an opportunity to contextualize the findings within existing literature and theoretical frameworks, contributing to the broader scholarly discourse on digital language education.

The combination of interviews and document analysis enhances the overall rigor of our study by providing a multifaceted approach to data collection and analysis. Interviews allow for the exploration of participants’ subjective experiences, emotions, and perceptions, providing rich qualitative data that offer insights into the nuances of language learners’ interactions with digital literacy, technostress, online engagement, autonomy, and academic success. On the other hand, document analysis adds depth and context to the findings by systematically reviewing academic records and online engagement metrics, providing data that complement and validate the qualitative insights gathered from interviews.

### Data Analysis procedures

The data analysis procedures for this study involved a thorough and systematic approach. The qualitative data, encompassing semi-structured interviews and document analysis, underwent manual coding to identify patterns, themes, and connections within the dataset. The coding process unfolded in multiple stages.

Initially, the recorded interviews were transcribed verbatim to ensure an accurate representation of participants’ responses. The transcriptions were systematically reviewed, and initial codes were assigned to pertinent segments of the data. This process was iterative, allowing codes to emerge organically from the data itself. Following initial coding, codes were organized into broader themes based on similarities and relationships.

Concurrently, document analysis entailed a meticulous examination of academic records and online engagement metrics. Relevant information was extracted, and codes were assigned to categorize and interpret the document data. This dual approach facilitated a comprehensive understanding of participants’ experiences.

To further enhance the credibility and dependability of the analysis, triangulation was employed by comparing and contrasting findings from different data sources. Peer debriefing and member checking were additional strategies employed to ensure the trustworthiness of the analysis. Peer debriefing involved discussions with colleagues to gain diverse perspectives, while member checking involved sharing summarized findings with participants to validate the accuracy and authenticity of their contributions.

The final stage of the analysis involved synthesizing the coded data into coherent narratives, offering a detailed and nuanced exploration of the relationships between digital literacy, technostress, online engagement, learner autonomy, and academic success among language learners in the online education context. Ethical considerations, including participant confidentiality and the responsible handling of data, were consistently upheld throughout the analysis process.

## Findings

### The Effect of Digital Literacy on Learners’ Technostress

The semi-structured interviews provided a rich source of opinions and experiences related to the effect of digital literacy on technostress among language learners in the online education context. Several participants expressed a sense of empowerment and reduced stress when navigating digital tools and platforms. For instance, one participant remarked on how their proficiency in utilizing online resources enhanced their overall learning experience, making the digital environment more accessible and less anxiety-inducing. In contrast, participants with lower levels of digital literacy often described feelings of frustration and stress, particularly when faced with unfamiliar technological interfaces and tools. These insights from the interviews underscored the varying impacts of digital literacy on technostress among the participants.

The document analysis of academic records and online engagement metrics provided quantitative support for the qualitative findings. Participants who demonstrated high levels of digital literacy, as evidenced by their active participation in online activities and successful academic performance, consistently reported lower instances of technostress. Conversely, participants with lower digital literacy levels exhibited signs of technostress, reflected in reduced engagement and academic performance metrics. These quantitative patterns aligned with the qualitative accounts, reinforcing the connection between digital literacy and technostress among language learners in the online education setting.

Peer debriefing sessions further enriched the interpretation of the opinions obtained from semi-structured interviews and document analysis. Colleagues corroborated the identified patterns, providing additional perspectives on the nuanced relationship between digital literacy and technostress. The discussions highlighted the significance of individual differences in interpreting and responding to the challenges posed by digital tools, emphasizing the need for tailored support and interventions to address technostress among language learners.

The opinions gathered from semi-structured interviews and document analysis collectively underscore the influence of digital literacy on technostress among language learners in the online education context. The interplay between individual experiences, proficiency in digital tools, and stress levels highlights the importance of fostering digital literacy skills to enhance the overall online learning experience.

Through the analysis of semi-structured interviews, document analysis, and peer debriefing, several themes emerged regarding the effect of digital literacy on technostress among language learners in the online education context.

1. **Empowerment and Confidence**: Participants consistently expressed a theme of empowerment and confidence associated with higher levels of digital literacy. Learners who felt proficient in using digital tools and platforms reported a sense of control over their online learning experiences. This empowerment was linked to reduced technostress, with learners expressing confidence in navigating the digital environment.
2. **Access and Adaptation Challenges**: A prevalent theme emerged among participants with lower levels of digital literacy, reflecting challenges related to access and adaptation. Learners highlighted difficulties in adapting to new technologies, expressing feelings of frustration and stress when faced with unfamiliar digital interfaces. Limited access to digital resources also contributed to heightened technostress for some participants.
3. **Positive Learning Experiences**: Participants with higher digital literacy levels consistently associated positive learning experiences with reduced technostress. Proficient use of online resources facilitated a smoother learning process, contributing to a positive online education experience. These learners demonstrated active engagement in online activities and exhibited higher levels of academic success.
4. **Frustration and Overwhelm**: Conversely, participants with lower digital literacy levels conveyed a theme of frustration and overwhelm. Challenges in using digital tools and platforms were linked to increased stress levels, impacting the overall online learning experience. This theme highlighted the importance of addressing digital literacy gaps to alleviate technostress among language learners.
5. **Individual Differences in Interpretation**: Peer debriefing discussions emphasized the theme of individual differences in interpreting and responding to technostress. Colleagues provided diverse perspectives on how learners perceive and cope with the challenges posed by digital tools. This theme underscored the need for personalized support and interventions to address the varied experiences of language learners.

These emergent themes collectively contribute to a nuanced understanding of the relationship between digital literacy and technostress, highlighting the multifaceted nature of learners’ experiences in the online education setting.

The Effect of Digital Literacy on Learners’ Online Engagement.

The semi-structured interviews offered a nuanced understanding of how digital literacy influenced online engagement among language learners in the online education context. Participants with advanced digital literacy demonstrated a heightened ability to actively engage with online learning activities. They expressed confidence in using digital tools to contribute to discussions, collaborate on tasks, and interact with course materials, fostering a more enriching online learning experience. On the contrary, participants with lower digital literacy levels faced challenges in fully participating in online activities, often feeling disconnected from the virtual learning community.

Document analysis, examining academic records and online engagement metrics, aligned with the qualitative findings. Learners with strong digital literacy skills consistently demonstrated higher rates of online engagement. Their documented participation in discussion forums, contributions to collaborative tasks, and effective utilization of digital resources highlighted the positive correlation between digital literacy and active online engagement. Conversely, participants with lower digital literacy levels exhibited lower rates of participation and interaction, indicating a direct impact on their online engagement metrics.

Peer debriefing sessions enriched the interpretation of these results by providing additional perspectives on the interplay between digital literacy and online engagement. Colleagues emphasized the coherence between qualitative and quantitative findings, reinforcing the significance of digital literacy in shaping learners’ online engagement experiences. Discussions underscored the importance of targeted interventions to enhance digital literacy skills, promoting improved online engagement among language learners.

The combined insights from semi-structured interviews, document analysis, and peer debriefing highlight the pivotal role of digital literacy in influencing online engagement dynamics. The findings emphasize the need for tailored strategies to address digital literacy gaps and enhance the overall online learning experience for language learners.

Several themes emerged from the analysis of semi-structured interviews, document analysis, and peer debriefing, shedding light on the nuanced relationship between digital literacy and online engagement among language learners in the online education context.

1. **Confidence and Active Participation**: Participants with higher levels of digital literacy consistently expressed a theme of confidence in actively participating in online learning activities. Proficiency in digital tools empowered learners to engage meaningfully in discussions, collaborate on tasks, and contribute effectively to the virtual learning community.
2. **Disconnected Learning Experience**: A recurring theme emerged among participants with lower digital literacy levels, reflecting a sense of disconnection from the online learning experience. Challenges in utilizing digital tools hindered their ability to actively engage in discussions and collaborative tasks, leading to a perceived lack of integration within the virtual learning community.
3. **Documented Participation Discrepancies**: Document analysis revealed a theme of documented participation discrepancies based on digital literacy levels. Learners with advanced digital literacy consistently demonstrated higher rates of participation in online engagement metrics, including discussion forum contributions and effective use of digital resources. Conversely, participants with lower digital literacy levels exhibited lower rates of participation and interaction.
4. **Impact on Learning Community Dynamics**: The findings highlighted a theme related to the impact of digital literacy on the dynamics of the online learning community. Learners with advanced digital literacy contributed positively to community interactions, fostering a collaborative and enriching virtual environment. In contrast, lower digital literacy levels were associated with challenges in actively participating, potentially influencing the overall community dynamics.
5. **Need for Targeted Interventions**: Discussions during peer debriefing sessions emphasized a theme regarding the need for targeted interventions. Colleagues recognized the significance of addressing digital literacy gaps to enhance online engagement among language learners. The theme underscored the importance of tailored strategies and support mechanisms to bridge digital literacy disparities and optimize the online learning experience.

In summary, these emergent themes collectively contribute to a comprehensive understanding of how digital literacy intricately shapes the online engagement experiences of language learners. The themes highlight the varying degrees of confidence, connectivity, and community dynamics influenced by learners’ digital literacy levels in the online education setting.

*The Effect of Digital Literacy on Learners’ Autonomy*.

The semi-structured interviews provided valuable insights into the influence of digital literacy on learners’ autonomy in the online education context. Participants with higher digital literacy levels expressed a greater sense of autonomy in managing their own learning processes. Proficiency in using digital tools allowed them to independently access information, navigate online resources, and engage in self-directed learning activities. These learners emphasized the role of digital literacy in empowering them to take control of their learning journey and make informed decisions regarding their educational pursuits.

Document analysis, examining academic records and online engagement metrics, reinforced the qualitative findings regarding the impact of digital literacy on learners’ autonomy. Learners with advanced digital literacy demonstrated a consistent pattern of self-directed learning, as reflected in their academic achievements and active engagement in online activities. The documented data highlighted the positive correlation between digital literacy and learners’ autonomy, emphasizing the role of technological proficiency in fostering independent learning behaviors.

Peer debriefing sessions enriched the interpretation of results by providing additional perspectives on the relationship between digital literacy and learners’ autonomy. Colleagues affirmed the coherence between qualitative and quantitative findings, acknowledging the role of digital literacy in shaping learners’ ability to navigate and control their learning experiences. Discussions underscored the need for educational interventions that enhance digital literacy skills to further promote learners’ autonomy in the online education landscape.

The combined insights from semi-structured interviews, document analysis, and peer debriefing consistently highlight the significant role of digital literacy in fostering learners’ autonomy. Higher levels of digital literacy empower learners to independently navigate online resources, engage in self-directed learning, and make informed decisions about their educational journey. These findings contribute valuable considerations for educators and institutions seeking to enhance learners’ autonomy in the digital learning environment.

Several themes emerged from the analysis of semi-structured interviews, document analysis, and peer debriefing, revealing the nuanced relationship between digital literacy and learners’ autonomy in the online education context.

1. **Empowerment through Digital Literacy**: Participants consistently highlighted a theme of empowerment associated with higher levels of digital literacy. Proficiency in using digital tools and navigating online resources empowered learners to take charge of their own learning processes. Digital literacy was seen as a catalyst for autonomy, providing learners with the skills and confidence to independently access information and engage in self-directed learning activities.
2. **Independence in Learning Activities**: A recurring theme was the independence demonstrated by learners with advanced digital literacy. These participants engaged in self-directed learning activities, independently exploring online resources, and managing their educational journey. The theme underscored the link between digital literacy and learners’ autonomy, emphasizing the role of technological proficiency in fostering independent learning behaviors.
3. **Documented Patterns of Autonomy**: Document analysis revealed a theme of documented patterns of autonomy based on digital literacy levels. Learners with higher digital literacy consistently exhibited self-directed learning behaviors, as evidenced by their academic achievements and active participation in online activities. The documented data reinforced the qualitative findings, highlighting the positive correlation between digital literacy and learners’ autonomy.
4. **Educational Interventions for Enhanced Autonomy**: Discussions during peer debriefing sessions emphasized a theme regarding the potential for educational interventions to enhance learners’ autonomy through digital literacy. Colleagues recognized the need for targeted strategies and interventions that focus on developing digital literacy skills to empower learners in navigating the complexities of the online learning environment.

In summary, these emergent themes collectively contribute to a nuanced understanding of how digital literacy influences learners’ autonomy. The themes highlight the empowering nature of digital literacy, its impact on independent learning activities, documented patterns of autonomy, and the potential for educational interventions to further enhance learners’ autonomy in the digital education landscape.

The Effect of Digital Literacy on Learners’ Academic Success.

The semi-structured interviews offered valuable insights into the impact of digital literacy on learners’ academic success in the online education context. Participants with advanced digital literacy consistently attributed their academic achievements to their proficiency in using digital tools and navigating online resources. These learners emphasized how digital literacy facilitated efficient information retrieval, enhanced study processes, and contributed to overall academic excellence. In contrast, participants with lower digital literacy levels expressed challenges in leveraging online resources effectively, which, in turn, affected their academic success.

Document analysis, examining academic records and online engagement metrics, provided quantitative support for the qualitative findings. Learners with higher levels of digital literacy demonstrated a pattern of academic success, as evidenced by higher grades, exam scores, and overall grade point averages. The documented data highlighted the positive correlation between digital literacy and academic success, emphasizing the role of technological proficiency in achieving positive educational outcomes.

Peer debriefing sessions enriched the interpretation of results by offering additional perspectives on the relationship between digital literacy and learners’ academic success. Colleagues concurred with the identified patterns, reinforcing the coherence between qualitative and quantitative findings. Discussions underscored the importance of considering digital literacy as a crucial factor influencing academic success in the context of online education.

The combined insights from semi-structured interviews, document analysis, and peer debriefing consistently point to the significant role of digital literacy in shaping learners’ academic success. Higher levels of digital literacy contribute to enhanced study processes, information retrieval, and overall academic excellence, highlighting the relevance of technological proficiency in achieving positive educational outcomes in the online learning environment.

Several themes emerged from the analysis of semi-structured interviews, document analysis, and peer debriefing, providing a comprehensive understanding of how digital literacy influences learners’ academic success in the online education context.

1. **Digital Literacy as a Catalyst for Academic Excellence**: Participants consistently emphasized a theme highlighting digital literacy as a catalyst for academic success. Learners with advanced digital literacy levels attributed their achievements to the efficient use of digital tools, navigation of online resources, and adept information retrieval. The theme underscored how digital literacy serves as a facilitator for academic excellence in the online learning environment.
2. **Challenges and Academic Impact for Lower Digital Literacy**: A recurring theme emerged among participants with lower digital literacy levels, indicating challenges in leveraging online resources effectively. These learners expressed difficulties in utilizing digital tools for academic purposes, which had a direct impact on their academic success. The theme highlighted the potential barriers posed by lower digital literacy skills in achieving positive educational outcomes.
3. **Quantitative Correlation between Digital Literacy and Academic Success**: Document analysis revealed a theme related to the quantitative correlation between digital literacy and academic success. Learners with higher levels of digital literacy consistently demonstrated patterns of academic success, as reflected in their grades, exam scores, and overall grade point averages. The theme emphasized the statistical evidence supporting the positive association between digital literacy and academic achievements.
4. **Recognition of Digital Literacy’s Importance in Academic Outcomes**: Discussions during peer debriefing sessions underscored a theme regarding the recognition of digital literacy’s importance in shaping academic outcomes. Colleagues acknowledged the consistent patterns identified in both qualitative and quantitative findings, affirming the critical role of digital literacy in contributing to learners’ academic success in the online education landscape.

In summary, these emergent themes collectively contribute to a nuanced understanding of how digital literacy influences learners’ academic success. The themes highlight digital literacy as a key facilitator for academic excellence, the challenges faced by learners with lower digital literacy, the quantitative correlation between digital literacy and academic success, and the widespread recognition of digital literacy’s significance in shaping positive educational outcomes.

In a nutshell, the themes that emerged from the analysis of semi-structured interviews, document analysis, and peer debriefing provide a comprehensive understanding of the intricate relationship between digital literacy and various aspects of language learners’ experiences in the online education context. Firstly, participants with higher digital literacy levels exhibited empowerment and confidence, leading to positive learning experiences and reduced technostress. Conversely, learners with lower digital literacy faced challenges in access and adaptation, resulting in frustration and overwhelm. Documented participation discrepancies based on digital literacy levels underscored the impact on learning community dynamics, while discussions highlighted the need for targeted interventions to bridge digital literacy gaps and optimize the online learning experience. These themes collectively emphasize the critical role of digital literacy in shaping online engagement, autonomy, and academic success among language learners.

## Discussion

The findings of this study unveil a nuanced interplay between digital literacy and the experiences of language learners in the online education domain. Digital literacy emerges as a critical determinant of learners’ engagement, autonomy, and academic success. The dual nature of digital literacy, acting as both an enabler and a potential source of stress, underscores the complexity of its role in shaping the online learning landscape.

Technostress, identified as a significant outcome, unveils the challenges language learners face in adapting to technology. The stressors, ranging from multitasking to ongoing technological changes, can impede engagement and contribute to a less favorable online learning experience. Understanding and addressing these technostress factors become imperative for educators and institutions aiming to create a supportive digital learning environment.

The study’s exploration of autonomy reveals a transformative potential linked to digital literacy. Learners proficient in digital tools exhibit a heightened sense of empowerment, navigating online resources independently. This aligns with broader educational goals of fostering autonomy and lifelong learning. Recognizing the empowering role of digital literacy can guide the development of strategies to cultivate independent learning behaviors in language education.

Academic success in the digital era is intimately tied to digital literacy proficiency. Learners adept in digital tools consistently demonstrate higher academic achievements, emphasizing the evolving landscape where technology proficiency is integral to effective learning. This finding calls for a reevaluation of traditional metrics and the incorporation of digital literacy into educational assessments to better capture the multifaceted nature of academic success.

In our study, an unexpected finding was the presence of technostress even among participants with high levels of digital literacy. Despite their proficiency in using digital tools, some learners still reported experiencing stress related to technology, which contradicted initial assumptions that digital literacy would serve as a protective factor against technostress. One potential reason behind this unexpected outcome could be the rapid pace of technological advancements, leading to constant updates and changes in digital platforms and tools. Even proficient users may encounter challenges when navigating new interfaces or features, resulting in feelings of frustration or anxiety. Additionally, individual differences in learning preferences and cognitive styles may influence how learners perceive and respond to technological challenges, highlighting the need for personalized support mechanisms tailored to address diverse technostress experiences. This unexpected finding underscores the complex and multifaceted nature of technostress, suggesting that digital literacy alone may not be sufficient to mitigate its effects in the dynamic landscape of online education.

The novelty of our study lies in its adoption of a phenomenological approach, offering a unique lens through which to explore the intricate dynamics of language learners’ experiences in the digital realm. While existing research has often employed quantitative methods to investigate the relationships between digital literacy, technostress, and academic success, our study ventures into uncharted territory by embracing a qualitative phenomenological design. This approach enables a deep and nuanced exploration of the lived experiences of language learners, allowing us to uncover the subjective meanings, emotions, and perceptions associated with the intersection of digital literacy, technostress, and academic success. By immersing ourselves in the rich narratives of language learners, we aim to provide a holistic understanding of the challenges and opportunities presented by the digital landscape, contributing novel insights that complement and expand upon existing quantitative studies in the field.

The findings of our study directly align with broader educational goals by emphasizing the importance of fostering digital literacy as a means of preparing learners for active participation in society. As highlighted in the theoretical background, digital literacy extends beyond mere technical skills and encompasses competencies crucial for engaging meaningfully in contemporary society. Our research demonstrates that learners proficient in digital tools exhibit a heightened sense of empowerment, autonomy, and engagement in online learning environments. By cultivating digital literacy skills among language learners, educators can equip them with the capabilities needed to navigate the digital landscape effectively, participate actively in online communities, and contribute meaningfully to society. Thus, our findings underscore the significance of integrating digital literacy initiatives into educational curricula as a means of fulfilling broader educational objectives aimed at preparing learners for success in the digital age and fostering their active engagement as informed citizens in an increasingly digitalized world.

The theoretical background lays the foundation for interpreting the study’s findings, particularly in the context of digital literacy. The results align with the concept of digital literacy as a complex, context-dependent skill set that evolves with technological advancements [22]. Language learners with advanced digital literacy skills demonstrated proficiency in navigating digital tools, emphasizing the importance of continually adapting to the changing technological landscape [26]. The study reinforces the broader notion that digital literacy goes beyond using technology for teaching; it involves acquiring competencies crucial for active participation in society [27].

The exploration of technostress resonates with Brod’s early characterization, highlighting the contemporary challenges arising from the use of emerging computer technologies [28]. The study expands on this concept, revealing how language learners experience stress due to various technological challenges, potentially affecting their well-being [36]. The findings underscore the need for addressing technostress in the educational sector, especially in the context of online assignments [35].

The study’s exploration of online engagement aligns with the multifaceted nature of the concept, encompassing diverse activities aimed at fostering meaningful interactions in the digital domain [11–12]. The identified components of online engagement, including behavioral, collaborative, and emotional aspects [12], contribute to the limited frameworks available for assessing engagement in higher education [37]. The study emphasizes the significance of online engagement in comprehending subject content.

Autonomy, as a foundational psychological need within the Self-Determination Theory [39–41], finds empirical support in the study. The results highlight the role of autonomy in influencing language learners’ engagement, persistence, and overall well-being. The study aligns with previous research, indicating autonomy as a more influential predictor of proficiency in online learning environments [46]. It acknowledges the potential of digital learning to enhance learners’ autonomy [49].

The concept of academic success, traditionally measured by grades and exam scores, has evolved in the digital age to encompass factors such as online participation and digital engagement [19]. The study’s findings, indicating a positive correlation between digital literacy skills and academic performance [20], resonate with the changing landscape of academic success. The study aligns with recent research suggesting that learners with higher digital literacy levels demonstrate greater adaptability to digital assessment methods, positively impacting overall academic success [21].

The current study’s findings both align and diverge from the empirical background, contributing to a nuanced understanding of the relationship between digital literacy, technostress, and academic success.

In contrast to [52], our study on language learners identified a nuanced interplay between digital literacy and technostress, shedding light on the challenges faced by students in online education. While [52] primarily focused on Chinese EFL students and utilized structural equation modeling (SEM), our study adopted a phenomenological approach, offering a qualitative exploration of language learners’ experiences, uncovering the multifaceted nature of technostress.

Similarly, [53] investigated the relationship between digital literacy, performance expectancy, and the intention to use digital technologies. Our study aligns with their emphasis on the interplay between digital literacy and various factors but delves into the specific context of language learners, revealing how technostress impacts online engagement, autonomy, and academic success.

In comparison to [55], which explored the relationships among students’ online engagement, digital technology attitude, digital literacy, and self-efficacy, our study emphasizes the negative impact of technostress on online engagement. While [55] highlighted the positive contributions of positive attitudes and digital literacy to self-efficacy, our findings suggest that technostress can hinder online engagement despite favorable attitudes and literacy levels.

Contrasting with [58], which investigated the impact of online education on digital literacy and autonomy readiness in a Turkish PST education program, our study focused on language learners. Despite both studies recognizing improvements in digital literacy and autonomy readiness post-online education, our findings reveal the nuanced challenges language learners face in achieving autonomy due to technostress.

Unlike [59], which studied the impact of digital literacy on the academic performance of university-level students, our study specifically examined language learners. While [59] highlighted the positive correlation between digital literacy and academic performance, our study adds depth by uncovering the role of technostress in shaping language learners’ academic success.

In comparison to [60], exploring the connection between digital literacy and academic performance in an online learning high school program, our study focuses on language learners in a broader context. While [60] proposed a three-stage model for academic performance, our findings contribute by emphasizing the nuanced challenges of technostress in language learners’ academic success.

Overall, our study enriches the existing literature by providing qualitative insights into the experiences of language learners in the digital realm, highlighting the intricate relationship between digital literacy, technostress, and academic success.

## Implications of the study

This study can have many implications for everybody involved in L2 learning. For teachers, our study offers valuable insights into the nuanced challenges that language learners face in the digital realm. Understanding the lived experiences of learners, as illuminated by our phenomenological approach, enables teachers to tailor their instructional strategies to better support students grappling with technostress and navigating the complexities of digital literacy. By fostering a more empathetic and informed teaching environment, educators can create interventions that address the specific needs of learners, promoting a healthier and more productive digital learning experience.

Materials developers can benefit from our study by gaining a deeper understanding of the factors influencing language learners’ engagement and autonomy in digital contexts. The qualitative insights garnered through phenomenological exploration shed light on the intricate relationship between digital literacy and learners’ experiences, informing the creation of materials that not only enhance digital skills but also alleviate technostress. Developing materials that resonate with the lived realities of language learners can contribute to more effective and engaging learning experiences.

Syllabus designers can draw on our findings to refine and adapt language learning curricula for the digital age. The phenomenological lens provides a nuanced understanding of the interplay between digital literacy and academic success, offering syllabus designers a foundation for integrating targeted interventions that promote autonomy and mitigate technostress. A curriculum informed by these insights can better prepare language learners to navigate the challenges of online education while capitalizing on the opportunities afforded by digital literacy.

Policy-makers can leverage our study to inform decisions related to the integration of technology in language education. Understanding the nuanced experiences of language learners in the digital realm is crucial for crafting policies that foster a supportive and conducive learning environment. Policymakers can use this qualitative understanding to implement measures that address technostress, promote digital literacy, and enhance overall academic success, contributing to the development of effective and learner-centered policies in the digital education landscape.

## Conclusion

In conclusion, our phenomenological study delved into the intricate dynamics of language learners’ experiences in the digital realm, focusing on the intersection of digital literacy, technostress, and academic success. By adopting a qualitative approach, we unearthed the subjective meanings, emotions, and perceptions associated with these phenomena, providing a rich and holistic understanding of the challenges and opportunities presented by the digital landscape. The narratives of language learners illuminated the multifaceted nature of technostress, the pivotal role of digital literacy in shaping online engagement and autonomy, and the nuanced impact of these factors on academic success.

Our study contributes to the existing body of knowledge by offering a unique perspective that complements quantitative studies in the field. The qualitative insights gained through phenomenological exploration provide depth and context to the complex relationships between digital literacy, technostress, and academic success. As a result, our findings have implications for educators, materials developers, syllabus designers, and policy-makers, offering practical insights for enhancing language learning experiences in the digital age.

It is paramount to recognize the ever-evolving nature of technology in the digital language learning landscape. The dynamic interplay between digital literacy, technostress, and academic success necessitates continuous exploration and adaptation to ensure effective educational practices in the online environment. As technology advances and new digital tools emerge, language educators, materials developers, syllabus designers, and policy-makers must remain vigilant in understanding the evolving dynamics and implications for language learners. Ongoing research endeavors are essential to stay abreast of emerging trends, challenges, and opportunities in digital language education. By fostering a culture of innovation and responsiveness to technological advancements, stakeholders can proactively address the evolving needs of language learners and promote their academic success in the 21st century digital era.

Moving forward, educators can use our study to inform pedagogical strategies that better support learners in navigating the digital landscape. Materials developers and syllabus designers can leverage the nuanced insights to create tailored resources that address the specific needs of language learners, promoting engagement, autonomy, and mitigating technostress. Additionally, policy-makers can utilize our findings to shape learner-centered policies that foster a positive and conducive environment for digital language education.

While our study sheds light on critical aspects of language learning in the digital era, it is important to acknowledge its limitations and the evolving nature of technology. Future research endeavors may delve deeper into specific dimensions uncovered in this study, further exploring the evolving landscape of digital literacy, technostress, and academic success. The dynamic interplay of these factors necessitates ongoing investigation to adapt educational practices to the ever-changing digital terrain and ensure the continued success of language learners in the 21st century.

## Data Availability

The dataset of the present study is available upon request from the corresponding author.
